# Trastuzumab-grafted PAMAM dendrimers for the selective delivery of anticancer drugs to HER2-positive breast cancer

**DOI:** 10.1038/srep23179

**Published:** 2016-04-07

**Authors:** Hitesh Kulhari, Deep Pooja, Shweta Shrivastava, Madhusudana Kuncha, V. G. M. Naidu, Vipul Bansal, Ramakrishna Sistla, David J. Adams

**Affiliations:** 1 Health Innovations Research Institute, RMIT University, Melbourne, VIC, 3083 Australia; 2 Ian Potter NanoBioSensing Facility, NanoBiotechnology Research Laboratory, School of Science, RMIT University, Melbourne, VIC, 3001, Australia; 3 IICT-RMIT Research Centre, CSIR-Indian Institute of Chemical Technology, Hyderabad, Telangana, 500607, India; 4 Medicinal Chemistry & Pharmacology Division, CSIR-Indian Institute of Chemical Technology, Hyderabad, Telangana, 500607, India; 5 Department of Pharmacology, National Institute of Pharmaceutical Education and Research, Hyderabad, India

## Abstract

Approximately 20% of breast cancer cases are human epidermal growth factor receptor 2 (HER2)-positive. This type of breast cancer is more aggressive and tends to reoccur more often than HER2-negative breast cancer. In this study, we synthesized trastuzumab (TZ)-grafted dendrimers to improve delivery of docetaxel (DTX) to HER2-positive breast cancer cells. Bioconjugation of TZ on the surface of dendrimers was performed using a heterocrosslinker, MAL-PEG-NHS. For imaging of cancer cells, dendrimers were also conjugated to fluorescein isothiocyanate. Comparative *in vitro* studies revealed that these targeted dendrimers were more selective, and had higher antiproliferation activity, towards HER2-positive MDA-MB-453 human breast cancer cells than HER2-negative MDA-MB-231 human breast cancer cells. When compared with unconjugated dendrimers, TZ-conjugated dendrimers also displayed higher cellular internalization and induction of apoptosis against MDA-MB-453 cells. Binding of TZ to the dendrimer surface could help site-specific delivery of DTX and reduce systemic toxicity resulting from its lack of specificity. In addition, *in vivo* studies revealed that the pharmacokinetic profile of DTX was significantly improved by the conjugated nanosystem.

In their GLOBOCAN 2012 report, the World Health Organization’s International Agency for Research on Cancer (IARC) states breast cancer is the second most common cancer diagnosed worldwide. In 2012, 1.7 million new cases were diagnosed, and more than 0.5 million women died from breast cancer. Since 2008, the incidence of breast cancer has increased by more than 20%, and mortality from the disease has increased by approximately 14%^1^,^2^. Around 20% of breast cancers are HER2-positive^3^. That is, they express high levels of the human epidermal growth factor receptor type 2 (HER2) that stimulates breast cancer cell growth. HER2-positive breast cancer is usually more aggressive and spreads more quickly than HER2-negative breast cancer. HER2-positive breast cancer can also be difficult to treat with the hormone therapies used for other types of breast cancer^3^,^4^.

Adjuvant and neo-adjuvant chemotherapies play important roles in managing HER2-positive breast cancer. Trastuzumab (TZ) is a humanized, monoclonal antibody approved by the Food and Drug Administration USA to treat HER2-positive early stage and metastatic breast cancer. It binds with HER2 receptors on breast cancer cells and blocks downstream signalling, leading to antibody-dependent cellular toxicity^4^,^5^,^6^,^7^.

In breast cancer, TZ can be used as a single agent and in combination with standard chemotherapy^8^. Combining TZ with other chemotherapeutic agents, such as taxanes and anthracyclines, has been seen to significantly improve disease-free survival rates and overall survival rates. When combined with docetaxel (DTX), TZ also shows synergistic effects^9^. The patients given a TZ-DTX combination treatment had a greater chance (p = 0.03) of survival (31.2 months) than people given DTX alone (22.7 months) but also suffered a higher incidence of febrile neutropenia and symptomatic heart failure^10^.

The specific binding of TZ to HER2 receptors has been explored to develop targeted systems for the imaging and delivery of anticancer drugs to HER2-positive breast cancer cells^11^,^12^. The presence of free functional groups (-NH_2_ and -COOH) on the surface of TZ provides the opppurtinity for conjugation of TZ to various drug carriers. TZ has been conjugated to different nanocarriers such as G4 poly(amido)amine (PAMAM) dendrimers, poly(lectic-co-glycolic acid), chitosan and albumin for targeted delivery of anticancer drugs and genes^12^,^13^,^14^,^15^,^16^.

DTX is a semi-synthetic paclitaxel analogue, and has been used as a first-line treatment for various cancers, including breast, lung, ovarian, brain and prostate cancers. Despite wide spectrum activity, its clinical application is restricted due its poor aqueous solibility, low bioavailability, non-specific toxicity and intolerable side effects^17^,^18^,^19^,^20^,^21^. Dendrimers have an ideal structure to develop a targeted drug delivery system where drug molecules can be encapsulated in the internal cavities while conjugating a targeting ligand to the free functional groups on the surface. Dendrimers are hyper-branched, nano-sized and multifunctional carrier systems having open internal cavities and free functional groups on their surface^22^,^23^,^24^,^25^,^26^,^27^.

Although reports detail conjugation of TZ to a nanocarrier system^28^,^29^,^30^,^31^,^32^, preclinical studies comparing the pharmacokinetic profile of conjugated system versus non-conjugated system and plain drug have not been reported. To this end, we have loaded DTX into amine terminated PAMAM dendrimers through non-covalent interactions and conjugated TZ to the dendrimers using a cross-linker MAL-PEG-NHS. This TZ grafted dendrimer-mediated drug delivery system was evaluated for controlled release of drug and site specific delivery of DTX to HER2 positive cancer cells. This study also aimed to elucidate the pharmacokinetic profile of the conjugated system.

## Results and Discussion

### Synthesis and characterization of trastuzumab-grafted PAMAM dendrimers

**Figure 1:**
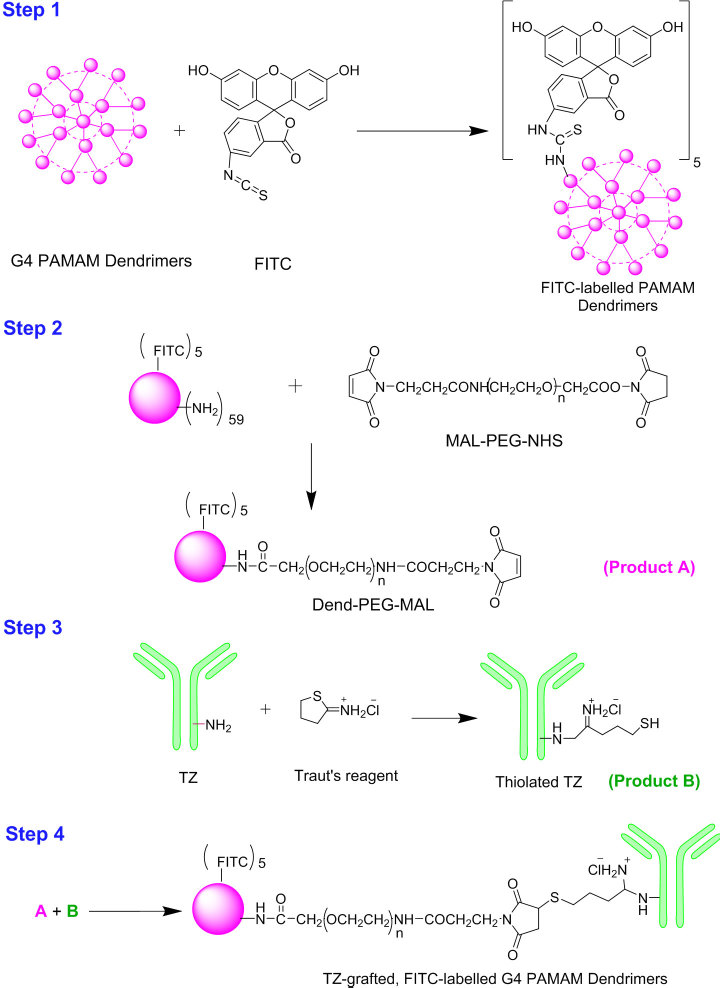
Schematic diagram illustrating the synthesis of Trastuzumab (TZ)-grafted, fluorescein isothiocyanate (FITC)-labelled G4 poly(amido) amine (PAMAM) dendrimers. In step 1, FITC was conjugated to G4 PAMAM dendrimers. In step 2, FITC-labelled dendrimers were crosslinked with MAL-PEG-NHS to give Dend-PEG-MAL (Product A). In step 3, TZ was thiolated using Traut’s reagent (2-iminothiolane) to give thiolated TZ. Finally, in step 4, Dend-PEG-MAL (Product A) was reacted to thiolated TZ (Product B) to synthesize TZ-grafted FITC-labelled G4 PAMAM dendrimers.

TZ was conjugated onto the surface of dendrimers by a four-step chemical reaction (Figure 1). Fluorescein isothiocyanate (FITC)-labelled dendrimers were synthesized by reacting the primary amino groups of the dendrimers with the FITC isothiocyanate groups. This reaction created a stable thiourea bond. UV/Vis spectrophotometric analysis revealed that approximately 4.6 FITC molecules were attached per dendrimer molecule (Fig. S1a). FITC-labelled dendrimers were crosslinked with the N-hydroxysuccinimide (NHS) ester of PEG-MAL. ^1^H NMR analysis confirmed pegylated dendrimer synthesis. Two new peaks appeared at 3.65 and 6.82 ppm, corresponding with PEG chain and MAL methylene group protons, respectively. This confirmed substantial conjugation between dendrimers and NHS-PEG-MAL (Fig. S1b).

In a separate reaction, TZ was thiolated using Traut’s reagent (2-iminothiolane hydrochloride) and the number of thiol groups on the TZ was determined using Ellmann’s test. Approximately 3.2 thiol groups were generated on per molecule of TZ. Thiolated TZ was then reacted with pegylated dendrimers, and conjugation confirmed by sodium dodecyl sulphate-polyacrylamide gel electrophoresis (SDS-PAGE) analysis (Fig. S1c).

Conjugation was also confirmed at various stages by studying the changes in surface charge of the dendrimers (Table 1). Due to the cationic 64-amine groups on their surface, plain dendrimers showed a positive zeta potential of 12.7 mV. After conjugation with NHS-PEG-MAL, the zeta potential decreased to 6.8 mV and, after TZ was grafted onto the dendrimer surface, it further reduced to −2.3 mV. Since TZ has an isoelectric point (pI) of 8.5, with an overall anionic nature at physiological pH, the reduction in zeta potential value supports successful conjugation of TZ with the dendrimers. The hydrodynamic diameter of PAMAM dendrimer, TZ-Dend and TZ-Dend-DTX was 12.8 ± 1.3 nm, 29.1 ± 3.9 nm and 31.6 ± 2.1 nm, respectively.

### DTX loading and *in vitro* drug release

DTX was loaded into plain dendrimers to a concentration of 241.7 ± 3.8 μg/mL. In TZ-Dend conjugates, the DTX loading concentration decreased significantly to 159.5 ± 5.4 μg/mL. This decrease may be due to presence of TZ on the dendrimer surface, which may restrict the DTX molecules’ access to the interior cavities. When dendrimers were conjugated with TZ after DTX loading, DTX concentration was 216.4 ± 2.79 μg/mL. In this case, the decrease in DTX loading (compared with plain dendrimers) may be due to loss or release of drug during the conjugation process. Since drug loading was higher when DTX loading occurred before TZ conjugation, we used this system in our further studies.

**Figure 2:**
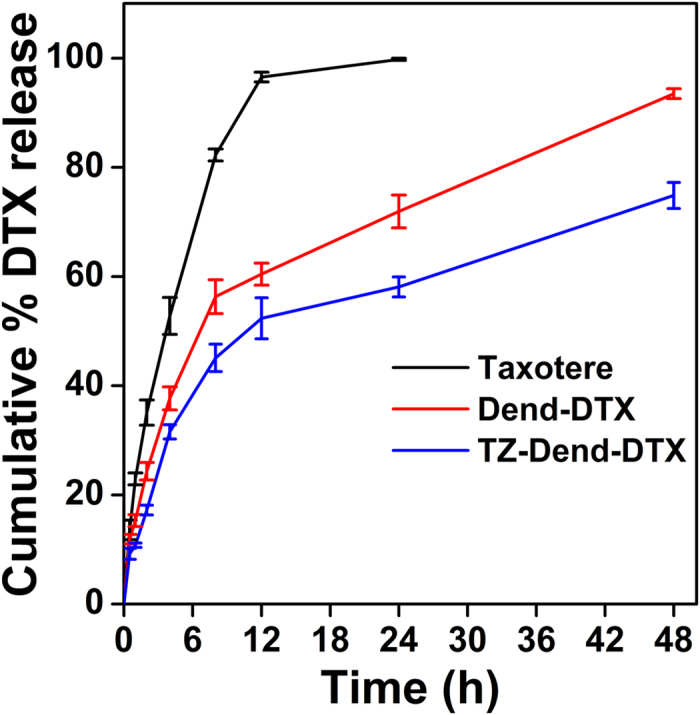
*In vitro* release of docetaxel (DTX) from three different formulations: Taxotere, DTX-loaded PAMAM dendrimers (Dend-DTX), and trastuzumab-conjugated Dend-DTX (TZ-Dend-DTX) in phosphate buffer saline (PBS) pH 7.4 (Mean ± SD; n = 3).

DTX release from Taxotere (a commercial DTX formulation), Dend-DTX and TZ-Dend-DTX was monitored in phosphate buffer saline (PBS) (Fig. 2). DTX was released faster from Taxotere than from dendrimer-mediated formulations. About 94% of DTX was released from Taxotere within 10 h. In contrast, Dend-DTX and TZ-Dend-DTX displayed controlled release of the drug over 48 h. Dend-DTX released 71.84% of drug after 24 h and 93.5% of drug after 48 h. TZ-Dend-DTX had better drug release properties than Dend-DTX or Taxotere alone, with DTX release at 58.6% after 24 h and 73.9% after 48 h. This slower release is likely due to the presence of TZ on the dendrimer surface, which could make a long release path for the drug or create a coat around the dendrimers that decreases the diffusion of drug from dendrimers.

### Hemolytic toxicity

**Figure 3:**
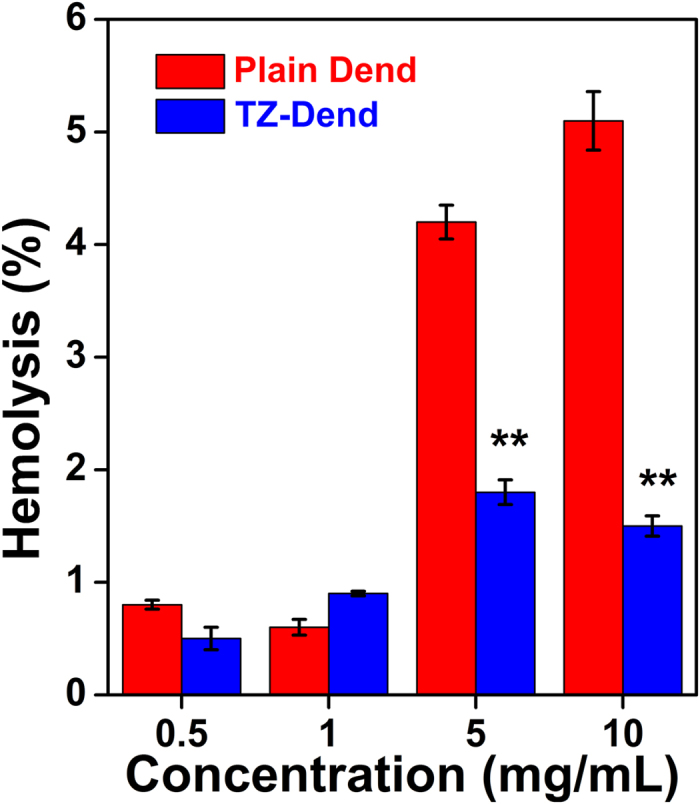
% Hemolysis caused by plain G4 PAMAM amine dendrimers (plain Dend) and trastuzumab-conjugated dendrimers (TZ-Dend) at various concentration levels (Mean ± SD; n = 3).

Biocompatibility of the drug delivery carrier was assessed by studying hemolytic toxicity. Plain dendrimers showed concentration-dependent hemolysis (Fig. 3). TZ-conjugated dendrimers caused lower hemolysis than plain dendrimers. At 10 mg/mL dendrimer concentration, plain dendrimers caused 5.3% hemolysis, compared with TZ-Dend conjugates which caused 1.5% hemolysis. These results suggest that TZ-Dend conjugates are more biocompatible than plain dendrimers with reduced hemolysis. The lower toxicity of TZ-Dend conjugates than plain dendrimers can be explained by the opposite charges on their surfaces (Table 1). Plain dendrimers have a high positive charge of 12.7 mV, whereas TZ-Dend conjugates were mildly anionic in nature, with a zeta potential value of −2.3 mV. PAMAM dendrimers cause hemolysis because of their cationic nature. They rapidly interact with the anionic membrane of red blood cells (RBC), causing hemolysis^33^. The TZ moiety on TZ-Dend conjugates neutralized this positive charge, making these conjugates slightly anionic, and decreasing the interaction between dendrimers and RBC membranes.

### *In vitro* cytotoxicity

Two breast cancer lines (MDA-MB-453 and MDA-MB-231) were used to investigate the anticancer actions of DTX formulations. These cell lines were chosen for their immuno-profiles. MDA-MB-453 cells are estrogen receptor-negative (ER^−^), progesterone receptor-negative (PR^−^) and HER2-positive. MDA-MB-231 cells are triple-negative (ER^−^, PR^−^ and HER2-negative). Moreover, both cell lines have a similar sensitivity to chemotherapy^34^,^35^. Therefore, a comparative evaluation of cytotoxicity against these cell lines could provide a good understanding of the contribution of TZ in targeting HER2-positive breast cancers.

**Figure 4:**
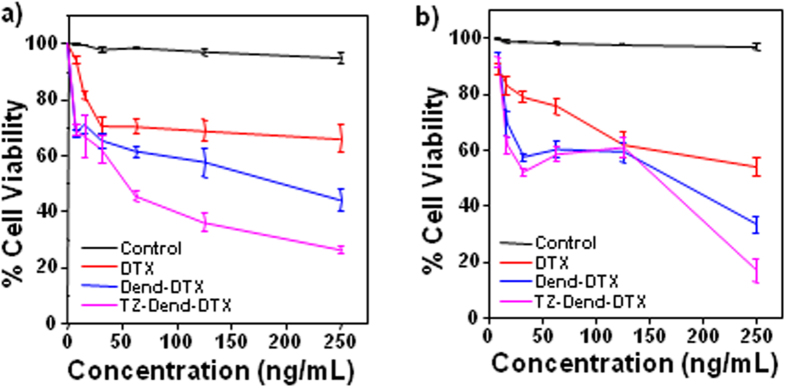
Percentage cell viability of (**a**) MDA-MB-453 and (**b**) MDA-MB-231 human breast cancer cells treated with varying concentrations of docetaxel (DTX), DTX-loaded PAMAM dendrimers (Dend-DTX) and trastuzumab-conjugated Dend-DTX (TZ-Dend-DTX) (Mean ± SD; n = 3). TZ-conjugated empty dendrimers were used as control.

Cytotoxicity measures of DTX, Dend-DTX and TZ-Dend-DTX against MDA-MB-453 and MDA-MB-231 cells are shown in Fig. 4a and Fig. 4b, respectively. At 125 ng/mL drug concentration, MDA-MB-453 cell viability after 48 h of treatment was 69.0, 57.6 and 36.2% for DTX, Dend-DTX and TZ-Dend-DTX, respectively. The results clearly indicated that DTX-loaded dendrimers were significantly more cytotoxic (p < 0.0005) than plain DTX. Comparing targeted and non-targeted dendrimers, TZ-Dend-DTX was more cytotoxic (p < 0.0005) than Dend-DTX in MDA-MB-453 cells. This may be due to the interaction between TZ and HER2 receptors in these cells, which makes internalization of TZ-Dend-DTX through receptor-mediated endocytosis more efficient. However, TZ-Dend-DTX did not cause significantly (p > 0.05) higher cytotoxicity than Dend-DTX in MDA-MB-231 cells. MDA-MB-231 cell viability after 48 h of treatment was 61.9, 59.3 and 60.9% for DTX, Dend-DTX and TZ-Dend-DTX, respectively (Fig. 4b).

**Figure 5:**
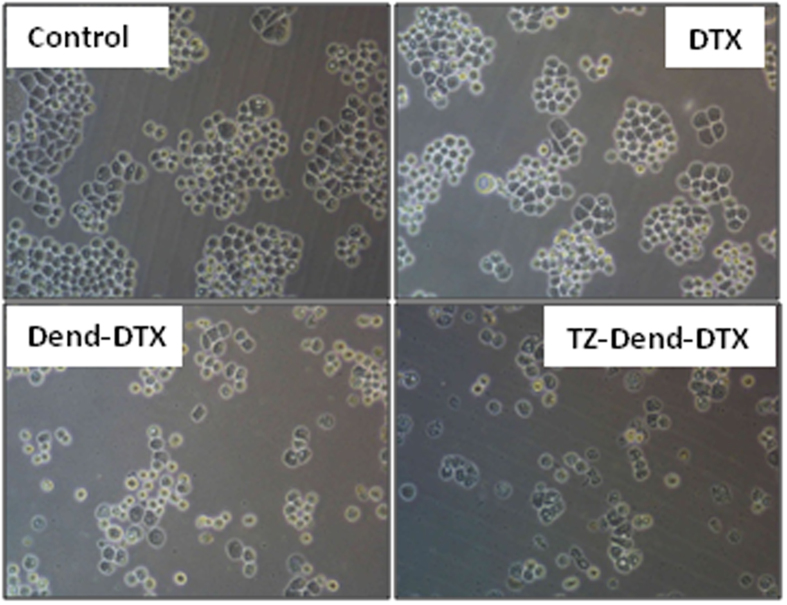
Morphology of MDA-MB-453 human breast cancer cells treated with 56 ng/mL of docetaxel (DTX), DTX-loaded PAMAM dendrimers (Dend-DTX) and trastuzumab-conjugated Dend-DTX (TZ-Dend-DTX).

All of the formulations also caused dose-dependent cytotoxicity in both cell lines. Cell viability decreased with increased drug concentrations. Against HER2-positive MDA-MB-453 cells, the half-maximal inhibitory concentration (IC_50_) values for Dend-DTX and TZ-Dend-DTX were 201 ng/mL and 56.18 ng/mL, respectively (Table S1). Therefore, the TZ-Dend-DTX conjugate was 3.57 times more cytotoxic than Dend-DTX. However, against HER2-negative MDA-MB-231 cells, the TZ-Dend-DTX conjugate was not significantly more effective than that of Dend-DTX. The IC_50_ value for Dend-DTX and TZ-Dend-DTX against MDA-MB-231 cells was 163.4 ng/mL and 149.5 ng/mL, respectively. Even at very low concentration (7.8 ng/mL), the TZ-Dend-DTX showed significantly (p < 0.05) higher cytotoxicity against HER2 positive MDA-MB-453 cells than Dend-DTX, but no significant difference (p > 0.05) in cytotoxicity against HER2 negative MDA-MB-231 cells. These results demonstrate that TZ can specifically target and deliver DTX to HER2-positive cells. After treatment with different DTX formulations, changes in cell morphology and a decrease in the number of viable MDA-MB-453 cells were observed (Fig. 5). TZ-conjugated empty dendrimers were used as a control and did not show significant toxicity.

### Cellular uptake

**Figure 6:**
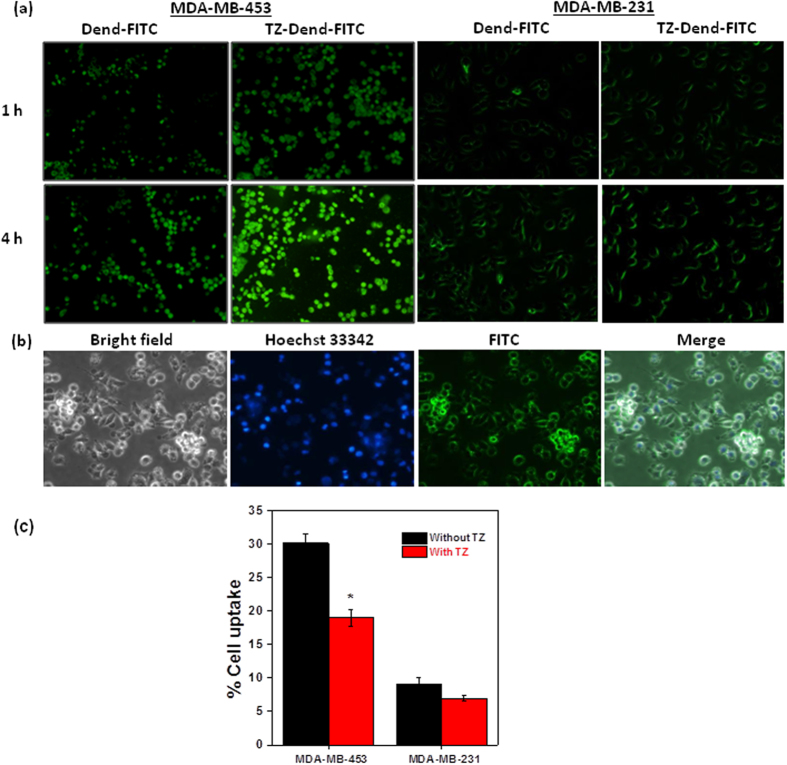
(**a**) Fluorescent microscopic images of MDA-MB-453 and MDA-MB-231 human breast cancer cells after 1 h and 4 h of incubation with FITC-conjugated dendrimers (Dend-FITC) and TZ-conjugated Dend-FITC (TZ-Dend-FITC); (**b**) Fluorescent microscopic images of cocultured MDA-MB-453 and MDA-MB-231 human breast cancer cells followed by incubation with TZ-Dend-FITC; (**c**) Quantitative uptake of TZ-Dend-FITC by MDA-MB-453 and MDA-MB-231 human breast cancer cells after 2 h incubation with or without TZ (20 mM).

Cellular uptake studies are important tools to track the internalization of fluorescent nanoparticles. Dend-FITC and TZ-Dend-FITC uptake was observed in both MDA-MB-453 (HER2-positive) and MDA-MB-231(HER2-negative) cells, and the results are shown in Fig. 6. In the case of MDA-MB-453, both Dend-FITC and TZ-Dend-FITC showed fluorescence after just 1 h. Fluorescence intensity was significantly higher after treatment with TZ-Dend-FITC than with Dend-FITC. These results were confirmed by quantitative analysis (Table 2). After 1 h of treatment, Dend-FITC and TZ-Dend-FITC uptake was 11.4% and 23.5%, respectively. Dendrimer formulations also showed time-dependent uptake. After 4 h of incubation, uptake increased to 34.2% and 57.9% for Dend-FITC and TZ-Dend-FITC, respectively. The significantly (p < 0.0005) higher uptake of TZ-Dend-FITC than Dend-FITC clearly demonstrates significance of TZ in the internalization of dendrimers in HER2-positive cells.

In contrast, both dendrimer formulations did not show any significant differences (p > 0.05) in cellular uptake in MDA-MB-231 which was also evident from quantitative data (Table 2). In order to confirm the selective targeting of TZ-Dend-FITC to HER2 positive cells, cellular uptake of TZ-Dend-FITC was observed in co-cultured (MDA-MB-453 and MDA-MB-231) cells. As shown in Fig. 6b, TZ-Dend-FITC was selectively uptaken by MDA-MB-453 cells (round in morphology). The competition assay demonstrated that the cellular uptake of TZ-Dend-FITC was significantly (p < 0.05) inhibited in the presence of TZ in MDA-MB-453 cells (Fig. 6c). These results revealed that the TZ conjugated dendrimers are uptaken more efficiently by HER2 overexpressing MDA-MB-453 cells.

### Qualitative determination of apoptosis with acridine orange and ethidium bromide staining

Acridine orange (AO) stains live and dead cells, whereas ethidium bromide (EB) stains only dead cells or cells that have lost membrane integrity. Therefore, staining is interpreted as follows:

- live cells: uniformly green with intact nuclei
- early apoptotic cells: green with intense green dots at the center due to chromatin condensation and nuclear fragmentation
- late apoptotic cells: orange with chromatin condensation and fragmented nuclei.

**Figure 7:**
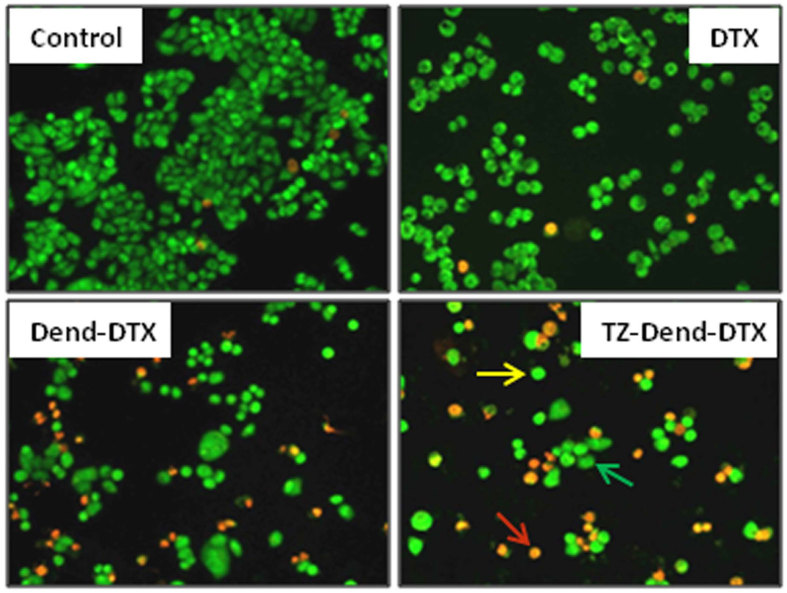
Qualitative determination of apoptosis induced by various DTX formulations, created with an AO/EB double-staining assay. Figure shows the fluorescent microscopic images of MDA-MB-453 human breast cancer cells after treatment with 56 ng/mL of docetaxel (DTX), DTX-loaded PAMAM dendrimers (Dend-DTX) and trastuzumab-conjugated Dend-DTX (TZ-Dend-DTX) for 24 h, then stained with 5 ng/mL each of AO and EB. Green, yellow and red arrows represent live cells, early apoptotic cells and late apoptotic cells, respectively.

Figure 7 shows the fluorescent images of untreated MDA-MB-453 cells, and cells treated with DTX, Dend-DTX and TZ-Dend-DTX after AO/EB double-staining. Untreated cells were an evenly spread green. Comparing the targeted and non-targeted dendrimers, cells treated with TZ-Dend-DTX showed greater apoptosis than those treated with Dend-DTX.

### Quantitative determination of apoptosis with an Annexin-V FITC/PI assay

**Figure 8:**
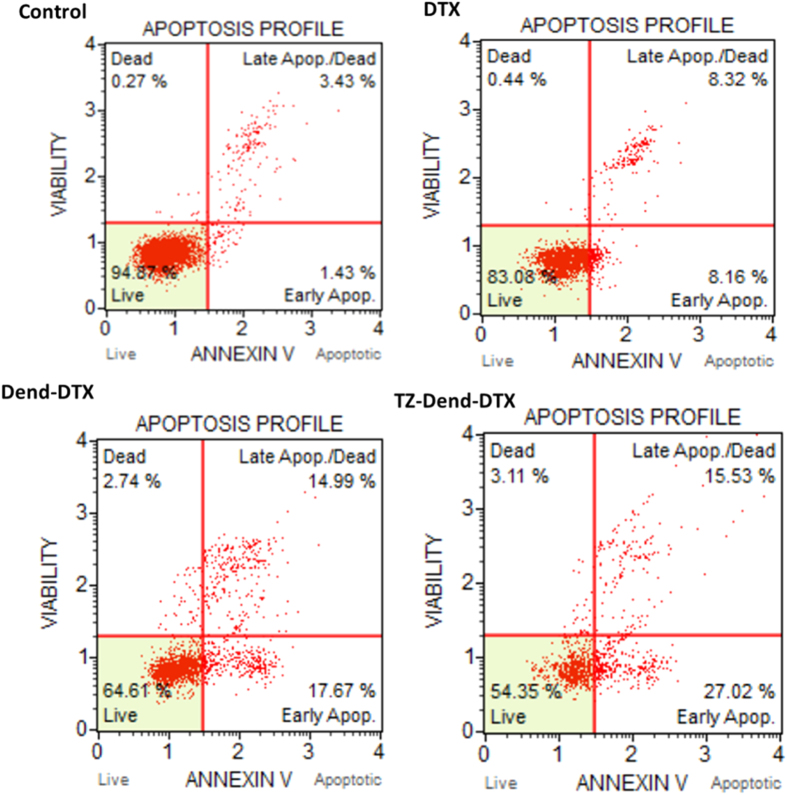
Quantitative determination of apoptosis induced by various DTX formulations, created with an annexin V FITC/PI assay. MDA-MB-453 human breast cancer cells were treated with 56 ng/mL of docetaxel (DTX), DTX-loaded PAMAM dendrimers (Dend-DTX) and trastuzumab-conjugated Dend-DTX (TZ-Dend-DTX) for 24 h.

Quantitation of apoptosis induced by different DTX formulations was studied with an Annexin-V FITC/PI assay (Fig. 8). A high-percentage of live cells (94.9%) was observed in untreated cells. The percentage of live cells decreased to 83.1% after DTX treatment, 64.6% after Dend-DTX treatment and 54.4% after TZ-Dend-DTX treatment. These data suggest that TZ-Dend-DTX induced more apoptosis than TZ-unconjugated and free DTX. The total percentage of apoptotic cells was 42.6%, 32.7% and 16.5% for TZ-Dend-DTX, Dend-DTX and DTX, respectively. Higher levels of apoptosis in TZ-conjugated dendrimers than unconjugated dendrimer-treated cells is likely due to an increased uptake and higher accumulation of DTX in these cells, which inhibits cellular proliferation.

### Pharmacokinetic studies

**Figure 9:**
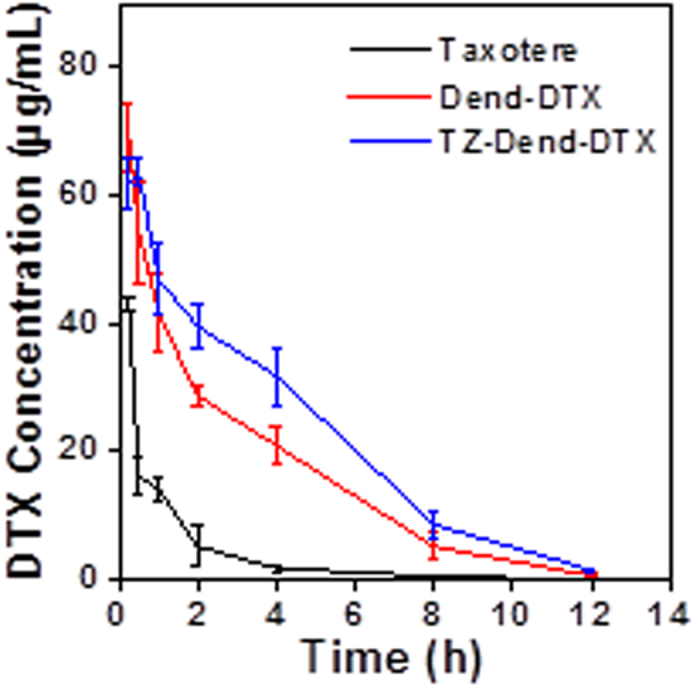
Time profiles of docetaxel (DTX) concentration in the plasma after intravenous administration of Taxotere, DTX-loaded G4 PAMAM amine dendrimers (Dend-DTX) and trastuzumab-conjugated Dend-DTX (TZ-Dend-DTX). Formulations were administered to BAlb/c mice at a dose of 10 mg/Kg, based on DTX. Data is expressed as Mean ± SD (n = 4).

Plasma-concentration profiles of DTX after intravenous injection of Taxotere, TZ-Dend-DTX and Dend-DTX are shown in Fig. 9 and correspond with the pharmacokinetic parameters in Table 3. Results suggest that the plasma pharmacokinetics of dendrimer conjugates (TZ-Dend-DTX and Dend-DTX) differed remarkably from that of Taxotere. The area-under-the-curve (AUC_0−∞_) of TZ-Dend-DTX (271.6 μg.h/mL) and Dend-DTX (204.2 μg.h/mL) was approximately 4.7 and 3.5 times higher, respectively, than that of Taxotere (57.2 μg.h/mL). This could be explained by the low amount of DTX released from dendrimer-based formulations. The observed clearance values for Taxotere, Dend-DTX and TZ-Dend-DTX were 268.8, 48.9 (5.5 times less) and 36.8 mL/h (7.3 times less), respectively. Mean residence time (MRT) increased by approximately 3.4 times and 2.5 times for TZ-Dend-DTX and Dend-DTX, respectively.

Compared with TZ-unconjugated dendrimers (Dend-DTX), TZ-Dend-DTX induced a significantly higher AUC_0−∞_ (271.6 versus 204.2, p < 0.005), lower clearance (36.8 versus 48.9, p < 0.05), and longer MRT values (6.85 versus 4.99; p < 0.05). The differences between the two dendrimer formulations may be attributed to the presence of PEG-linkers and TZ on the surface of TZ-Dend-DTX. PEG is a well-documented molecule that could alter the pharmacokinetics of different nanocarrier systems^36^,^37^,^38^,^39^. Being an antifouling molecule, PEG increases the circulation time of nanoparticles in the blood stream by delaying their capture by the reticulo-endothelial system. Additionally, TZ is a high molecular weight, clinically approved mAb (~145 KD), known for its high specificity for HER2 receptors^40^. It may therefore lengthen TZ-Dend-DTX circulation and slow release of DTX from conjugated dendrimers.

### Stability studies

After two months of storage of TZ-Dend-DTX in the refrigerator at 4 °C, there was a slight change in consistency, however no signs of precipitation or significant change in zeta potential and drug content was observed. (Table 4).

In summary, multifunctional TZ-conjugated dendrimers were synthesized using a heterocross-linker MAL-PEG-NHS and characterized by DLS, NMR and PAGE analysis. Targeted dendrimers selectively and specifically targeted DTX to HER2-positive breast cancer cells. Targeted TZ-Dend-DTX inhibited the growth of HER2-positive cancer cells to a greater extent than non-targeted Dend-DTX, as demonstrated by our anti-proliferation and apoptosis studies. Furthermore, grafting TZ onto the surface of dendrimers decreased the hemolytic toxicity of unconjugated cationic PAMAM dendrimers and enhanced the circulation half-life of the conjugate. These findings could help to develop a better therapeutic profile for DTX and deliver better health outcomes for people with HER2-positive cancer.

## Methods

### Materials

G4 PAMAM dendrimers (MW: 14215 Da) with a diaminobutane core were purchased from NanoSynthons (Mt Pleasant, MI). TZ was purchased from Nava Sanjivani Drugs (Hyderabad, India). MAL-PEG-NHS (MW: 865.92 Da) was purchased from Thermo Fisher Scientific. DTX was a gift from TherDose Pharma Pvt Ltd (Hyderabad, India). High-performance liquid chromatography (HPLC) grade solvents were purchased from Merck specialties (Mumbai, India). Nylon membrane filters with a pore size of 0.22 μm were obtained from Pall India Pvt Ltd (Mumbai, India). FITC, dialysis tubing (molecular weight cut off 2000), Dulbecco’s modified eagle medium (DMEM), trypsin–EDTA, antibiotic/anti-mycotic solution, PBS (Ca^2+^, Mg^2+^ free), 3-(4, 5- dimethylthiazol-2-yl)-2, 5-diphenyl tetrazolium bromide (MTT), dimethyl sulfoxide (DMSO), annexin V-FITC apoptosis detection kit, AO and EB were purchased from Sigma-Aldrich (St. Louis, MO, USA). Fetal bovine serum was purchased from Gibco, USA. Cell culture plastic ware was purchased from Tarson Ltd (Mumbai, India).

### Synthesis and characterization of FITC-labelled, TZ-conjugated PAMAM dendrimers

FITC-labelled, TZ-conjugated PAMAM dendrimers were synthesized in a four-step chemical reaction:

1. synthesis of FITC-labelled dendrimers
2. conjugation of heterocross-linker to FITC-labelled dendrimers
3. TZ thiolation
4. bioconjugation of thiolated TZ with dendrimers.

#### Synthesis of FITC-labelled dendrimers

FITC-labelled G4 PAMAM dendrimers were prepared using a previously reported method with slight modifications^41^. Dendrimers were dissolved in PBS (pH 7.4) and mixed with FITC solution in 1:6 molar ratio. The mixture was stirred overnight, then dialyzed against PBS for 24 h to remove unconjugated or free FITC. The molar ratio of FITC conjugation to dendrimers was determined from absorbance at 495 nm using a UV-VIS spectrophotometer. For qualitative analysis, samples were scanned in the wavelength-range of 200–800 nm. The FITC-labelled dendrimer solution was lyophilized and used for further reactions.

#### Conjugation of heterocross-linker, Maleimide-poly(ethylene) glycol-N-hydroxysuccinimide (NHS-PEG-MAL) to FITC-labelled dendrimers

NHS ester of PEG-MAL was reacted with FITC-conjugated dendrimer in a molar ratio of 3:1 in PBS (pH 8) and stirred for 30 minutes at room temperature. Dend-PEG-MAL synthesis was characterized by ^1^H NMR spectroscopy. Dend-PEG-MAL conjugate was dissolved in D_2_O and scanned using Avance 500 NMR.

#### TZ thiolation

We dissolved TZ in PBS, and reacted with 2-iminothiolane hydrochloride in 1:10 molar ratio for 2 h. Thiolated TZ was purified by dialysis (MW cut-off 12000–14000) in PBS to remove the 2-iminothiolane hydrochloride.

#### Bioconjugation of thiolated TZ with dendrimers (TZ-Dend)

Thiolated TZ was coupled with heterocross-linked, conjugated, FITC-labelled dendrimers (10:1 ratio) to give a bioconjugate. The NHS-PEG-MAL cross-linked dendrimer solution was mixed with the thiolated TZ solution and allowed to react at room temperature overnight. TZ-Dend conjugate was purified using size exclusion chromatography (Sephadex column G-25 M) and eluted with PBS, pH 7.4.

### Characterization of TZ-Dend conjugate

The surface potential of various nanoconjugates was measured using a Malvern Zetasizer Nano-ZS (Malvern Instruments Ltd, UK). Samples were diluted in deionized water and analyzed at 25 °C. TZ-Dend conjugates were also characterized by PAGE analysis. All gels were electrophoresed under reducing conditions using Mini-Protean II electrophoresis units from BioRad at a constant voltage of 200 V in Tris/glycine/SDS buffer. The hydrodynamic diameter of PAMAM dendrimers, TZ-Dend and TZ-Dend-DTX was determined using a Malvern Zetasizer Nano-ZS (Malvern Instruments Ltd., Malvern, Worcestershire, UK).

### Drug loading

An excess (5 mg) of DTX was added to the dendrimer solution, sonicated for 30 sec, and gently stirred overnight. Two approaches were used to prepare TZ-Dend-DTX formulations. In first method, DTX-loaded dendrimers were conjugated with TZ. In the second method, DTX was loaded into Dend-TZ conjugates. Formulations were equilibrated for 24 h, then filtered through a 0.45 μm nylon membrane filter. The concentration of DTX was determined by HPLC, as per our previously validated method^18^.

### *In vitro* drug release studies

*In vitro* drug release was studied using a dialysis method in PBS (pH 7.4). Taxotere and dendrimer formulations, equivalent to 2 mg of DTX, were placed in dialysis tubing (MWCO 2000) in release medium (100 mL of PBS) at 37 °C and stirred at 100 rpm. At different time intervals, an aliquot of 1 mL was withdrawn from the release medium and replaced with the same volume of fresh medium. The samples were appropriately diluted, filtered through a 0.22 μm nylon filter, and analyzed with HPLC for DTX content.

### Cell culture

Human breast cancer cell lines MDA-MB-453 (HER2-positive) and MDA-MB-231 (HER2-negative) were obtained from the American Type Culture Collection (ATCC) (Manassas, VA, USA). Cells were grown in DMEM medium supplemented with 10% fetal bovine serum, 100 U/mL penicillin, 100 mg/mL streptomycin and 2 mM/L L-glutamine. Cells were maintained at 37 °C in 5% CO_2_.

### Anti-proliferation assay

Cells were seeded in 96-well plates at a density of 5 × 10^3^ cells per well in 100 μL of medium. They were allowed to adhere overnight before being incubated with DTX, or Dend-DTX or TZ-Dend-DTX, at equivalent drug concentrations ranging from 5–250 ng/mL for 48 h. Media were then replaced with serum-free DMEM containing MTT (0.5 mg/mL), then cells were incubated for 4 h. Media were removed and 150 μL of DMSO was added to dissolve formazan crystals. Absorbance was measured at 570 nm using a microplate reader. Untreated cells were used as a negative control (100% viability). IC_50_ was calculated by fitting the curve of cell viability against the drug concentration.

### Cellular uptake studies

For qualitative uptake studies, cells were seeded in 12-well plates. After 24 h, cells were incubated with Dend-FITC or TZ-Dend-FITC conjugates, at an equivalent FITC concentration (100 μg/mL). For competitive binding studies, cells were preincubated with an excess (20 mM) of TZ for 2 h and then treated with TZ-Dend-FITC. Culture media was removed at pre-determined time intervals. Cells were then rinsed twice with cold PBS and observed by fluorescence microscopy. For selective cellular uptake studies, both MDA-MB-453 and MDA-MB-231 cells were cocultured and treated with TZ-Dend-FITC conjugate. For quantitative studies, 0.1% Triton X-100 in 0.2 M NaOH was added to each well to lyse cells. Fluorescence intensities were measured using a microplate reader at an excitation wavelength of 495 nm and emission wavelength of 520 nm.

### AO/EB double-staining assay

AO/EB assays were used to visualize changes in cell morphology, such as chromatin condensation and apoptotic body formation, which are characteristic of apoptosis. MDA-MB-453 cells were seeded in six-well plates at 5 × 10^5^ cells per well, in 2 mL of DMEM, and cultured for 24 h. Cells were incubated with various DTX formulations (DTX, Dend-DTX or TZ-Dend-DTX), equivalent to 56 ng/mL of DTX. After 24 h, the medium was removed and cells were washed twice in PBS before being incubated in PBS-containing AO and EB (5 μg/mL of each) at 37 °C for 10 min. Cell viability was observed with a fluorescence microscope.

### Apoptosis

To evaluate apoptosis and necrosis, we used Annexin V-FITC/PI assays, as per the manufacturer’s protocols. Briefly, MDA-MB-453 cells (1 × 10^6^ cells/well) were seeded in six-well plates and maintained at 37 °C in 5% CO_2_ and a 95% humidified atmosphere. The medium was replaced with freshly prepared medium containing drug-loaded formulations. Untreated cells were used as controls. After 24 h, cells were washed twice with PBS and resuspended in 500 μL of binding buffer. We added 5 μL of FITC-conjugated Annexin V and 10 μL of PI, and incubated cells for 15 min at room temperature in the dark. Cells were then analyzed by a Muse^™^ Cell Analyzer (Merck-Millipore, Germany) and the extent of apoptosis and necrosis was determined.

### Pharmacokinetic studies

Female Balb/c mice were purchased from the National Institute of Nutrition (Hyderabad, India). The Institutional Animal Ethics Committee (IAEC) of the CSIR-Indian Institute of Chemical Technology, Hyderabad, approved all experimental protocols for the study. All animal studies were performed in accordance with the guidelines of the Committee for the Purpose of Control and Supervision of Experiments on Animals (CPCSEA). Animals were kept in polypropylene cages under standard laboratory conditions (12 h light, 12 h dark cycle).

Eighty-four female mice (20–25 g) were randomly assigned to three groups for pharmacokinetic investigations. Taxotere, Dend-DTX or TZ-Dend-DTX was injected via the tail vein at an equivalent dose of 10 mg/kg DTX. At scheduled time intervals (0.25, 0.5, 1, 2, 4, 8 and 12 h) post-injection, 0.3 mL of blood was collected into heparinized polyethylene tubes from the retro-orbital plexus and centrifuged at 1500 rpm for 5 min to obtain plasma. The samples were stored at −80 °C until they were analyzed.

The analytical method for determining the DTX content of plasma samples was validated using an HPLC system (Water, USA) equipped with a C18 column (Grace, 250 × 4.6 mm, 5 μm) and a photodiode array detector^42^. The mobile phase consisted of acetonitrile (48%) and 0.1% orthophosphoric acid (52%). 5 μL of paclitaxel solution (1 mg/mL) was used as an internal standard. The flow rate was maintained at 1 mL/min, and the column temperature was set at 25 ± 5 °C. Peaks were monitored at a wavelength of 230 nm. Retention times for DTX and PTX were 9.1 min and 11.2 min, respectively.

### Stability studies

The final formulation (TZ-Dend-DTX) was stored at 4 °C for 60 days. The formulation was monitored for any changes in consistency and precipitation, and analyzed for zeta potential and drug content.

### Statistical analysis

Results are expressed as the mean and standard deviation of three experiments (n = 3). Pharmacokinetic data are an average of four experiments (n = 4) and pharmacokinetic parameters were calculated using WinNonlin^®^ software (Certara USA Inc., Princeton, NJ). Statistical significance was analyzed using the student t-test for two groups and one-way ANOVA for multiple groups. A probability (p) of less than 0.05 was considered as statistically significant.

## Additional Information

**How to cite this article**: Kulhari, H. *et al*. Trastuzumab-grafted PAMAM dendrimers for the selective delivery of anticancer drugs to HER2-positive breast cancer. *Sci. Rep.* **6**, 23179; doi: 10.1038/srep23179 (2016).

## Supplementary Material

Supplementary Information

## Figures and Tables

**Table 1:** Zeta potential value of plain dendrimers, dendrimer-PEG-NHS ester and dendrimer-Trastuzumab conjugate (Dend-TZ).

Step No.	Conjugate	Zeta Potential (mV)
1	Dendrimers	12.3 ± 0.9
2	Dendrimer PEG-NHS ester	6.8 ± 1.5
3	Dend-TZ conjugate	−2.3 ± 0.6

**Table 2:** Cellular uptake of Dend-FITC and TZ-Dend-FITC by human breast cancer cells MDA-MB-453 and MDA-MB-231 after 1 and 4 h of incubation.

Formulation	MDA-MB-453		MDA-MB-231	
	% Cell uptakeafter 1 h	% Cell uptakeafter 4 h	% Cell uptakeafter 1 h	% Cell uptakeafter 4 h
Dend-FITC	11.28 ± 1.4	34.2 ± 1.95	5.39 ± 2.6	12.57 ± 3.1
TZ-Dend-FITC	23.49 ± 1.85***	57.92 ± 2.71***	8.65 ± 1.9^ns^	17.06 ± 2.4^ns^

*Indicates comparison with Dend-DTX (ns Not Significant, ***p < 0.0005).

**Table 3:** Pharmacokinetic parameters for the three DTX formulations-Taxotere, DTX loaded dendrimers (Dend-DTX) and Trastuzumab grafted Dend-DTX (TZ-Dend-DTX).

PK Parameter	Taxotere	Dend-DTX	TZ-Dend-DTX
C_max_ (μg/mL)	42.7 ± 1.82	69.01 ± 4.38**	61.72 ± 3.92^**,ns^
T_1/2_ (h)	1.35 ± 0.21	1.99 ± 0.3^*^	3.24 ± 0.17^*,#^
Cl (mL/h)	268.81 ± 11.2	48.97 ± 5.65^**^	36.82 ± 4.06^**,#^
AUC_0−∞_	37.2 ± 2.94	204.16 ± 8.03^***^	271.58 ± 7.95^***,##^
MRT (h)	1.98 ± 0.08	4.99 ± 0.19^**^	6.85 ± 0.11^***,##^

*Indicates comparison with Taxotere; # Indicates comparison with Dend-DTX.

Statistical analysis: ns Not Significant, *p < 0.05, **p < 0.005, ***p < 0.0005, ^#^*p* < 0.05, ^##^*p* < 0.005.

**Table 4:** Physicochemical stability of TZ-Dend-DTX conjugate after 60 days of storage in refrigeration conditions (4 °C).

Parameter	Day 0	Day 60
Precipitation	No	No
Change in consistency	No	+
ZP (mV)	−2.3 ± 0.72	−3.89 ± 0.51
% DTX content	100	99.12
