# Supplementary material for: Trastuzumab-grafted PAMAM dendrimers for the selective delivery of anticancer drugs to HER2-positive breast cancer
Source: Sci Rep. 2016 Apr 7;6:23179. doi: 10.1038/srep23179 (PMC4823704; doi:10.1038/srep23179)

Supporting information

Trastuzumab-grafted PAMAM dendrimers for the selective delivery of anticancer drugs to HER2-positive breast cancer

Hitesh Kulhari[1,2,3,4], Deep Pooja[4], Shweta Shrivastava[5], Madhusudan Kuncha[4], V.G.M. Naidu[5], Vipul Bansal[1,2], Ramakrishna Sistla[4,*] and David J. Adams[1,*],

[1]Health Innovations Research Institute, RMIT University, Melbourne, VIC, 3083 Australia

[2]Ian Potter NanoBioSensing Facility, NanoBiotechnology Research Laboratory, School of Applied Sciences, RMIT University, Melbourne, VIC, 3001, Australia

[3]IICT-RMIT Research Centre, CSIR-Indian Institute of Chemical Technology, Hyderabad, Telangana, 500607, India

[4]Medicinal Chemistry & Pharmacology Division, CSIR-Indian Institute of Chemical Technology, Hyderabad, Telangana, 500607, India

[5]Department of Pharmacology, National Institute of Pharmaceutical Education and Research, Hyderabad, India.

**Email: david.adams@rmit.edu.au (D.J.A.); sistla@iict.res.in (R.S.)*

<u>D.J. Adams current affiliation</u>: Illawarra Health & Medical Research Institute (IHMRI), University of Wollongong, Wollongong, NSW 2522 Australia. Email: djadams@uow.edu.au

Table S1. Half-maximal inhibitory concentration (IC_{50}) values for different formulations of docetaxel against human breast cancer cells MDA-MB-453 and MDA-MB-231 after 48 h of incubation.

**Formulation**	**IC_{50} values (ng/mL)**	
	**MDA-MB-453**	**MDA-MB-231**
DTX	> 250	> 250
Dend-DTX	201 ± 4.2	163.4 ± 3.8
TZ-Dend-DTX	56.2 ± 2.9	149.5 ± 4.6

Figure S1a. UV/Vis absorbance spectra of plain G4 PAMAM dendrimers, fluorescein isothiocynate (FITC) and FITC-conjugated G4 PAMAM dendrimers (Dend-FITC).

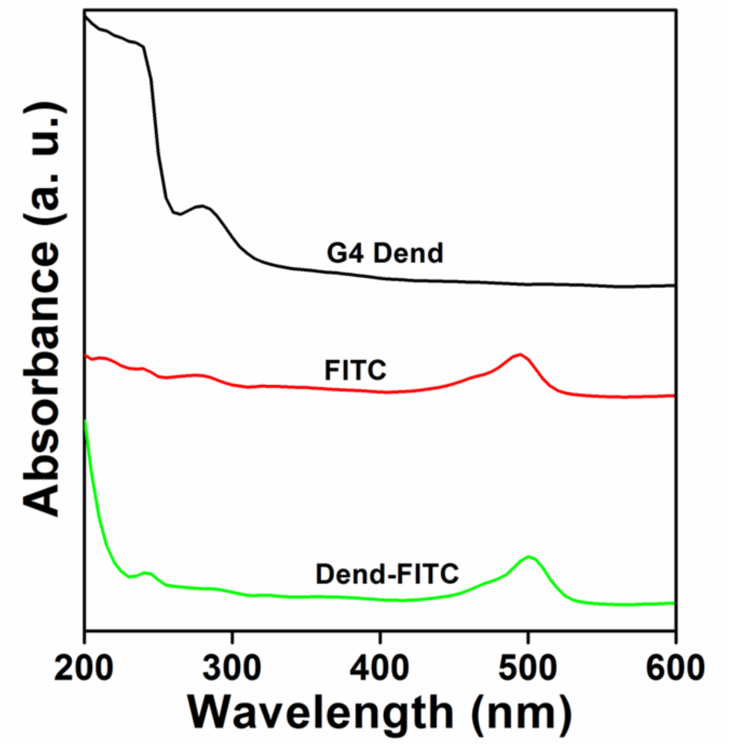

Figure S1b. ^{1}H NMR spectra of G4 PAMAM dendrimers and PEGylated G4 PAMAM dendrimers.

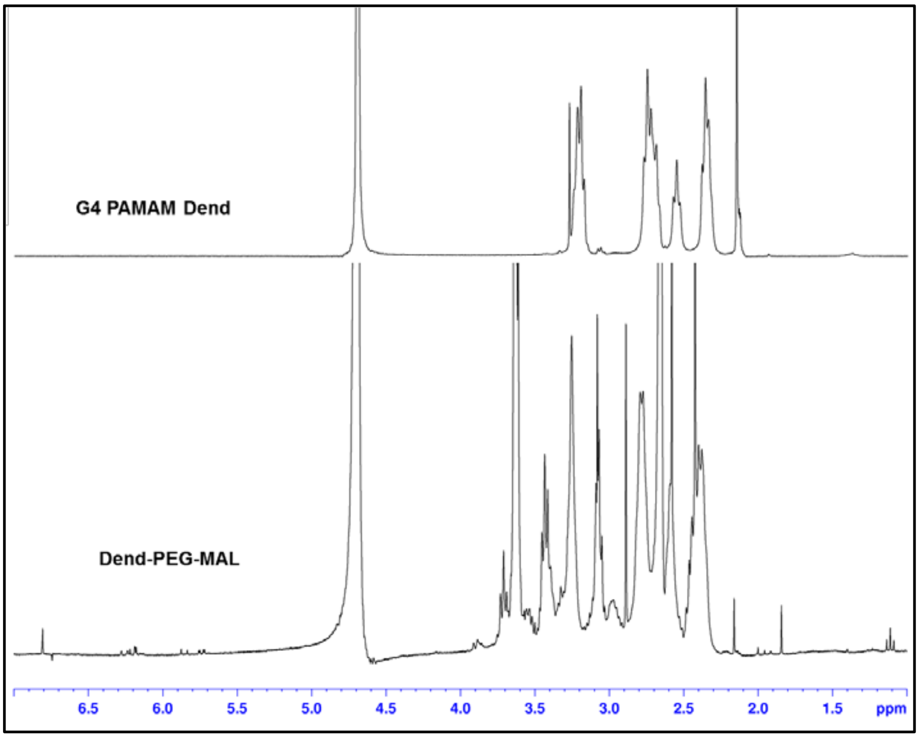

Figure S1c. Polyacrylamide gel electrophoresis of G4 PAMAM dendrimers (Dend), trastuzumab (TZ) and TZ-conjugated dendrimers (Dend-TZ). The reduced mobility of a number of bands along with a band streaking in Dend-TZ above those in the TZ alone, indicates successful conjugation of TZ with dendrimers. The cropped area of the gel is shown as dotted lines. The electrophoretic gel for the test compounds (Dend, TZ and Dend-TZ) and marker (M) have been run under the same experimental conditions (at a constant voltage of 200 V in Tris/glycine/SDS buffer).

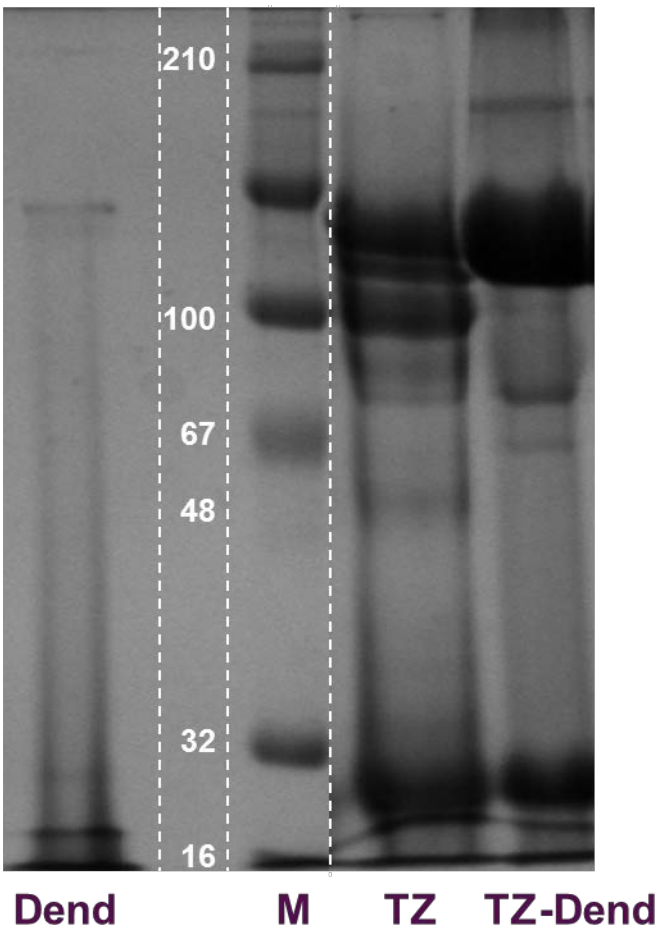

Supplement: Supplementary Information [file srep23179-s1.pdf]
